# Supplementary material for: Acute Blood Pressure Changes Following Resistance Exercise in Adults with Hypertension
Source: J Funct Morphol Kinesiol. 2025 Sep 12;10(3):349. doi: 10.3390/jfmk10030349 (PMC12452289; doi:10.3390/jfmk10030349)
Supplement: Supplementary file 1 [file jfmk-10-00349-s001.zip › jfmk-3810241-supplementary.pdf]

Table S1. Supplementary file. Simple linear regression between blood pressure delta changes and independent variable.

Condition	Exercise	Dependent variables	Independent variables	r^2	B	p-values
HIGH INTENSITY	SBP	Squat	Sex	0.082	-8.20	0.156
			BMI	0.000	0.79	0.922
			Age	0.002	-0.61	0.841
			ARB	0.240	-4.46	0.477
			Diuretic	0.000	-0.75	0.94
			Calcio	12.25	0.57	0.238
			ACE	0.006	3.02	0.697
			Beta	0.006	-5.40	0.711
		Row	Sex	0.045	-4.56	0.296
			BMI	0.00	-0.01	0.993
			Age	0.04	-0.22	0.327
			ARB	0.039	-220	0.334
			Diuretic	0.079	10.8	0.163
			Calcio	0.028	6.42	0.412
			ACE	0.006	2.27	0.695
			Beta	0.820	-15.2	0.156
		Deadlift	Sex	0.210	3.21	0.477
			BMI	0.042	-0.62	0.316
			Age	0.036	0.22	0.357
			ARB	0.082	-6.30	0.157
			Diuretic	0.006	2.96	0.075
			Calcio	0.065	10.0	0.210
			ACE	0.033	5.30	0.373
			Beta	0.008	-4.96	0.6659
		Bench Press	Sex	0.100	-2.50	0.634
			BMI	0.100	-1.11	0.116
			Age	0.003	-0.08	0.784
			ARB	0.29	-4.39	0.404
			Diuretic	0.06	11.2	0.229
			Calcio	0.008	4.13	0.661
			ACE	0.004	2.14	0.759
			Beta	0.025	-10.1	0.438
LOW INTENSITY	SBP	Squat	Sex	0.006	-1.82	0.700
			BMI	0.005	0.23	0.722
			Age	0.000	0.02	0.925

			ARB	0.001	0.73	0.878
			Diuretic	0.053	9.42	0.260
			Calcio	0	0.20	0.98
			ACE	0.056	-7.16	0.246
			Beta	0.002	2.28	0.846
		Row	Sex	0.008	-1.56	0.67
			BMI	0.034	-0.45	0.37
			Age	0.023	-0.13	0.464
			ARB	0.006	-1.37	0.704
			Diuretic	0.084	9.17	0.151
			Calcio	0	6.46	0.939
			ACE	0	0.25	0.959
			Beta	0.063	-10.9	0.218
		Deadlift	Sex	0.001	-0.75	0.879
			BMI	0.057	-0.79	0.241
			Age	0.018	-0.17	0.509
			ARB	0.035	-4.49	0.36
			Diuretic	0.169	17.6	0.037*
			Calcio	0	0.29	0.974
			ACE	0.01	-3.22	0.62
			Beta	0.006	4.44	0.717
		Bench Press	Sex	0.053	-4.77	0.258
			BMI	0.057	-0.68	0.241
			Age	0.107	-0.35	0.103
			ARB	0.053	-4.77	0.258
			Diuretic	0.137	13.67	0.063
			Calcio	0.011	3.92	0.606
			ACE	0	-0.46	0.936
			Beta	0.003	-3.00	0.776
HIGH INTENSITY	DBP	Squat	Sex	0.022	-2.06	0.475
			BMI	0.03	0.32	0.398
			Age	0.046	0.15	0.292
			ARB	0.092	-4.26	0.132
			Diuretic	0.022	3.75	0.467
			Calcio	0.133	9.17	0.067
			ACE	0.004	1.14	0.766
			Beta	0.006	-2.64	0.713
		Row	Sex	0.99	-4.22	0.117
			ARB	0.001	-0.48	0.861
			BMI	0.044	0.33	0.303

			Age	0.013	0.07	0.581
			Diuretic	0.002	4.92	0.314
			Calcio	0.101	7.63	0.113
			ACE	0.133	-6.46	0.067
			Beta	0.002	1.60	0.815
		Deadlift	Sex	0.087	-3.38	0.143
			BMI	0.044	0.33	0.303
			Age	0.13	0.67	0.581
			ARB	0.031	-2.02	0.389
			Diuretic	0.02	2.88	0.493
			Calcio	0.028	3.42	0.415
			ACE	0.008	1.36	0.661
			Beta	0.025	-4.52	0.436
		Bench Press	Sex	0.043	-2.68	0.311
			BMI	0.119	-0.21	0.562
			Age	0.04	0.03	0.845
			ARB	0.133	-4.72	0.067
			Diuretic	0.17	9.54	0.036*
			Calcio	0.17	9.54	0.036*
			ACE	0.002	-0.82	0.816
			Beta	0.023	-4.88	0.458
LOW INTENSITY	DBP	Squat	Sex	0.02	2.29	0.49
			BMI	0.085	-0.19	0.68
			Age	0.195	0.16	0.34
			ARB	0.14	-6.04	0.06
			Diuretic	0.024	4.46	0.451
			Calcio	0.003	-1.50	0.801
			ACE	0.073	5.75	0.182
			Beta	0.076	11.0	0.172
		Row	Sex	0	0.09	0.977
			BMI	0	-0.01	0.99
			Age	0.022	0.12	0.472
			ARB	0.047	-3.31	0.288
			Diuretic	0.038	5.29	0.342
			Calcio	0.001	-0.67	0.906
			ACE	0.029	3.41	0.408
			Beta	0	0.06	0.934
		Deadlift	Sex	0.017	2.62	0.524
			BMI	0.089	-0.82	0.138
			Age	0.052	0.05	0.801

	ARB	0.238	-9.78	0.011*
	Diuretic	0.01	3.50	0.635
	Calcio	0	0.79	0.915
	ACE	0.17	10.9	0.036*
	Beta	0.071	9.76	0.187
Bench Press	Sex	0.011	-1.48	0.618
	BMI	0.059	-0.48	0.233
	Age	0.014	0.09	0.564
	ARB	0.187	-6.75	0.016*
	Diuretic	0.004	5.23	0.747
	Calcio	0.023	3.88	0.462
	ACE	0.155	7.48	0.046*
	Beta	0.014	4.24	0.562

* $p<0.05$, association significative
